# Prediction of violent reoffending in prisoners and individuals on probation: a Dutch validation study (OxRec)

**DOI:** 10.1038/s41598-018-37539-x

**Published:** 2019-01-29

**Authors:** Seena Fazel, Achim Wolf, Maria D. L. A. Vazquez-Montes, Thomas R. Fanshawe

**Affiliations:** 1 Department of Psychiatry, Warneford Hospital, University of Oxford, Oxford, UK; 2 Nuffield Department of Primary Care Health Sciences, University of Oxford, Oxford, UK

## Abstract

Scalable and transparent methods for risk assessment are increasingly required in criminal justice to inform decisions about sentencing, release, parole, and probation. However, few such approaches exist and their validation in external settings is typically lacking. A total national sample of all offenders (9072 released from prisoners and 6329 individuals on probation) from 2011–2012 in the Netherlands were followed up for violent and any reoffending over 2 years. The sample was mostly male (n = 574 [6%] were female prisoners and n = 784 [12%] were female probationers), and median ages were 30 in the prison sample and 34 in those on probation. Predictors for a scalable risk assessment tool (OxRec) were extracted from a routinely collected dataset used by criminal justice agencies, and outcomes from official criminal registers. OxRec’s predictive performance in terms of discrimination and calibration was tested. Reoffending rates in the Dutch prisoner cohort were 16% for 2-year violent reoffending and 44% for 2-year any reoffending, with lower rates in the probation sample. Discrimination as measured by the c-index was moderate, at 0.68 (95% CI: 0.66–0.70) for 2-year violent reoffending in prisoners and between 0.65 and 0.68 for other outcomes and the probation sample. The model required recalibration, after which calibration performance was adequate (e.g. calibration in the large was 1.0 for all scenarios). A recalibrated model for OxRec can be used in the Netherlands for individuals released from prison and individuals on probation to stratify their risk of future violent and any reoffending. The approach that we outline can be considered for external validations of criminal justice and clinical risk models.

## Introduction

Risk assessment tools in criminal justice, forensic mental health, and clinical psychiatry are increasingly used to stratify individuals into different categories based on their predicted future risk of crime and violence. In criminal justice, such tools are variously used to inform decision-making at sentencing, release, parole, and probation. In clinical settings, such tools are used less frequently, and assist in determining treatment, discharge timing and conditions, particularly in forensic psychiatry, and also the need for further assessments^1^. The extent to which the use of these tools have improved outcomes is uncertain, with only one randomised controlled trial to date in outpatients that reported that criminal outcomes were no different, and violent crime outcomes worse, in settings that added a structured clinical judgement tool to routine violence risk assessment^2^. Nevertheless, many criminal justice and mental health systems have adopted these approaches as one way to provide more consistency to their assessments, improve transparency, and inform treatment and management decision. One recent international survey of more than 2000 mental health professionals reported their regular use in 44 countries with more than 200 individual tools^3^.

However, these tools are typically time-consuming and associated with substantial direct and indirect costs. For example, one study found that a typical risk assessment in forensic psychiatry consumes 16 person-hours^4^. The most widely used risk assessment in clinical settings, the Historical Clinical Risk Management-20 (HCR-20)^1^, requires regular training that is typically a few days in duration and that usually costs hundreds of dollars^5^. In criminal justice, many tools used are also time-consuming, and some jurisdictions purchase assessment tools from commercial organizations. A trend has been for these tools to evaluate apparent criminogenic needs. At the same time, the conflation of risk and needs might detract from the predictive accuracy of these tools in that some of the strongest risk factors may not be modifiable in the way that criminogenic needs are usually considered, and some needs may not be associated with reoffending risk. In addition, some of these tools do not have prespecified risk categories, and thus what high risk actually means varies substantially^6^. Due to these problems, simple scalable tools have been developed, which do not require specific training, are free to use, and can be completed within half an hour, including two for inpatient violence in psychiatry (Broset^7^ and DASA^8^), one in severe mental illness for violent crime (OxMIV)^9^, and another for use in released prisoners to predict violent reoffending (OxRec)^10^.

One of the key issues with current approaches is their performance in real world settings. For example, the widely used HRC-20^11^ and PCL-R^12^, have been recently demonstrated to have poor predictive validity in field studies, also known as shrinkage in performance, in contrast to their validity in research studies with small samples. For many other tools, no information exists on their external validation. The poor performance of some of these tools in practice is partly a consequence of their development in different samples from the ones in which they are being used. In addition, the researchers developing older tools have used methods that are now considered low quality. For example, prespecifying factors and outcomes being investigated, statistical power (e.g. having at least 10 outcome events per predictor in derivation studies^13^ and 100 outcome events in validation studies^14^), and using multivariable regression to test the incremental value of individual factors have rarely been implemented^15^.

One tool in criminal justice that follows these methods is the Oxford Risk of Recidivism Tool, OxRec^10^. It was developed and externally validated in Sweden using a total population of prisoners, and provides both a probability score for violent reoffending and stratifies according to prespecified low, medium and high categories. It represents a considerable advance in criminal justice because it takes around 10–15 minutes to complete, relies on mostly routinely collected information, has an online calculator that can be used freely by mental health and criminal justice professionals, does not require any formal training, and performs as well as current approaches to risk assessment in criminal justice that take many hours^16^. Its discrimination was moderately high compared with other risk assessment instruments in criminal justice^16^ – an overall area under the curve of 0.76 for 2 year violent reoffending in an external validation sample of more than 14,000 prisoners, with sensitivity and specificity of 67% and 70%, respectively, and positive and negative predictive values of 37% and 89%, respectively. A key strength was that OxRec was developed using a prespecified protocol, which outlined what and how predictor variables would be tested and categorized before any statistical analyses were conducted. Nevertheless, external validations outside Sweden have not been performed, and are required to test its performance in different settings. Thus, we have conducted an external validation on a total cohort of all offenders in the Netherlands over a two-year period.

## Results

Data from risk assessments performed in 2011 and 2012 were available for 9072 prisoners and 6329 non-prisoners. There were some differences in individual characteristics between the Dutch prisoner and non-prisoner cohorts, and also differences from the Swedish cohort (Table 1). In particular, compared to the Dutch prisoner cohort, the non-prisoner cohort had a larger female proportion (12% vs 6%), were of similar age (median 34 vs 30 years), and had a higher prevalence of a violent index (most recent) offence (65% vs 54%). Compared to the Swedish cohort, the Dutch prisoner cohort had slightly lower median age (30 vs 36 years) and a higher prevalence of a previous violent crime conviction (67% vs 53%), violent index offence (54% vs 38%) and drug use (30% vs 23%). Among the variables with a lower weighting in the OxRec tool, there were also some differences in education and income classification as a result of differences in how these variables were defined. Missing values of risk factors across the whole cohort were infrequent (no more than 10% for any risk factor, Table 1).

**Table 1:** Distribution of risk factors for the two Dutch cohorts, and comparison with the original Swedish cohort.

Variable	Sample of prisoners (n = 9072)		Sample of non-prisoners (n = 6329)		Comparison with Fazel *et al*.^10^
	Summary	Missing data	Summary	Missing data	
Sex – Female	574 (6%)	5 (0.06%)	784 (12%)	1 (0.02%)	7%
Age	Median 30	—	Median 34	—	Median 36
	IQR 23 to 41		IQR 24 to 44		IQR 27 to 46
Immigrant	Not available		Not available		31%
**Length of incarceration**					
<6 months	6938 (76%)	172 (2%)	Not applicable		69%
6–12 months	1043 (11%)				16%
12–24 months	560 (6%)				10%
>=24 months	359 (4%)				4%
Violent index offence	4913 (54%)	26 (0.3%)	4081 (65%)	15 (0.2%)	38%
Previous violent crime	6050 (67%)	—	3116 (49%)	—	53%
Civil status – Unmarried	6783 (75%)	697 (8%)	4301 (68%)	495 (8%)	65%
**Education**					
Only primary or special education	1693 (19%)	327 (4%)	843 (13%)	294 (5%)	48%
No secondary diploma	3277 (36%)		1638 (26%)		46%
Secondary diploma (age 16–22)	3775 (42%)		3554 (56%)		6%
Employment	2308 (25%)	348 (4%)	3245 (51%)	241 (4%)	25%
**Income**					
“Low”	6572 (72%)	378 (4%)	3601 (57%)	273 (4%)	53%
“Medium”	2122 (23%)		2455 (39%)		40%
Deprivation	Median 0.59	910 (10%)	Median 0.37	342 (5%)	Median 0.39
	IQR −0.23 to 1.57		IQR −0.33 to 1.30		IQR −1.18 to 1.47
Alcohol use	1947 (21%)	322 (4%)	1113 (18%)	272 (4%)	22%
Drug use	2697 (30%)	338 (4%)	1006 (16%)	276 (4%)	23%
Any mental disorder	2448 (27%)	677 (7%)	1561 (35%)	415 (7%)	22%
Any severe mental disorder	Not available		Not available		3%

**Figure 1:**
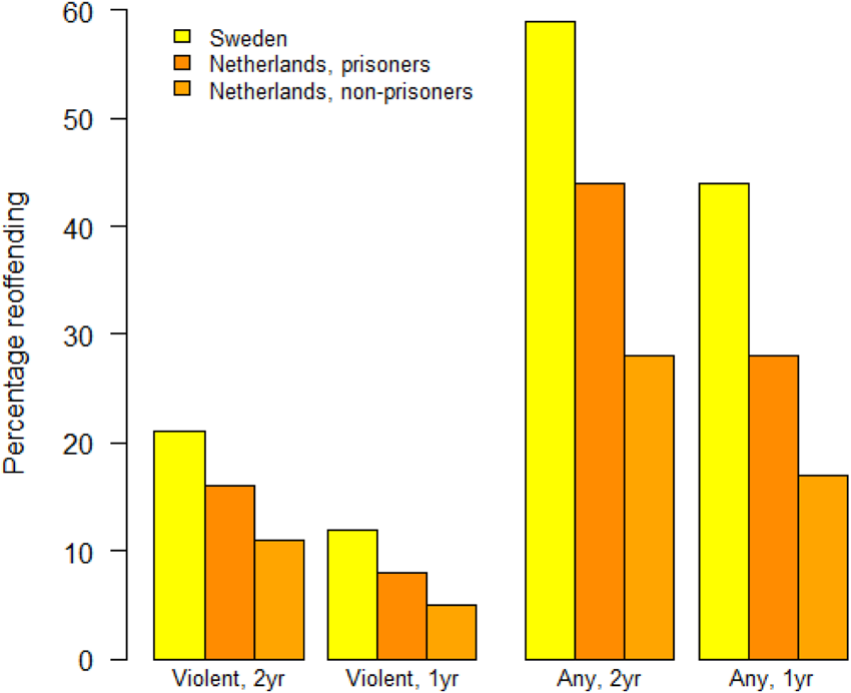
Comparison of reoffending rates in the two Dutch cohorts and the original Swedish cohort.

Reoffending rates in the Dutch prisoner cohort were around one third lower than in the Swedish cohort (2-year violent reoffending 16% [Dutch] vs. 21% [Swedish], 2-year any reoffending 44% vs. 59%), and were lower still in the Dutch non-prisoner cohort (Fig. 1 and Supplementary Table 2).

**Figure 2:**
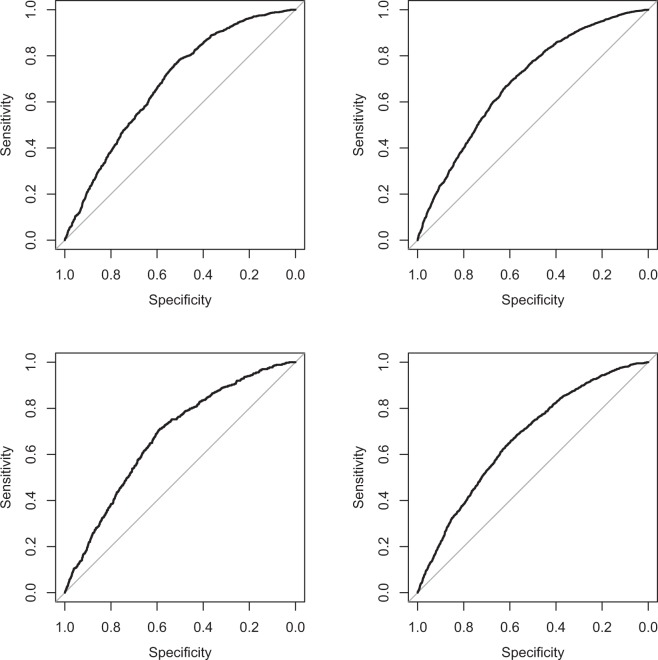
Receiver operating characteristic curve for 2-year violent reoffending in Dutch prisoners and non-prisoners. Note: Upper left: 2-year violent reoffending, prisoners. Upper right: 2-year any reoffending, prisoners. Lower left: 2-year violent reoffending, non-prisoners. Lower right: 2-year any reoffending, non-prisoners.

In the Dutch validation of OxRec, discrimination as measured by the c-index was moderate, at 0.68 (95% CI: 0.66–0.70) for 2-year violent reoffending in prisoners (Fig. 2) and was very similar for other outcomes (between 0.65 and 0.68 for 1-year/2-year, violent/any reoffending, prisoners/non-prisoners; Fig. 2 for 2-year outcomes; Supplementary Fig. 1 for 1-year outcomes). As the reoffending rate was lower than in the Swedish cohort (Supplementary Table 2), the predicted numbers of outcome events using the uncalibrated OxRec were much higher than the numbers that were observed in the Dutch sample (Supplementary Table 3), which meant that the calibration using the existing OxRec tool was suboptimal. This was apparent for all outcomes, but was particularly pronounced for those in non-prisoners, for whom the incidence of reoffending was much lower (Supplementary Table 2). There was a close relationship between the ratio of predicted to observed events and the observed event rate ratio of the Swedish to Dutch cohorts, suggesting that recalibration of the OxRec tool was necessary.

**Figure 3:**
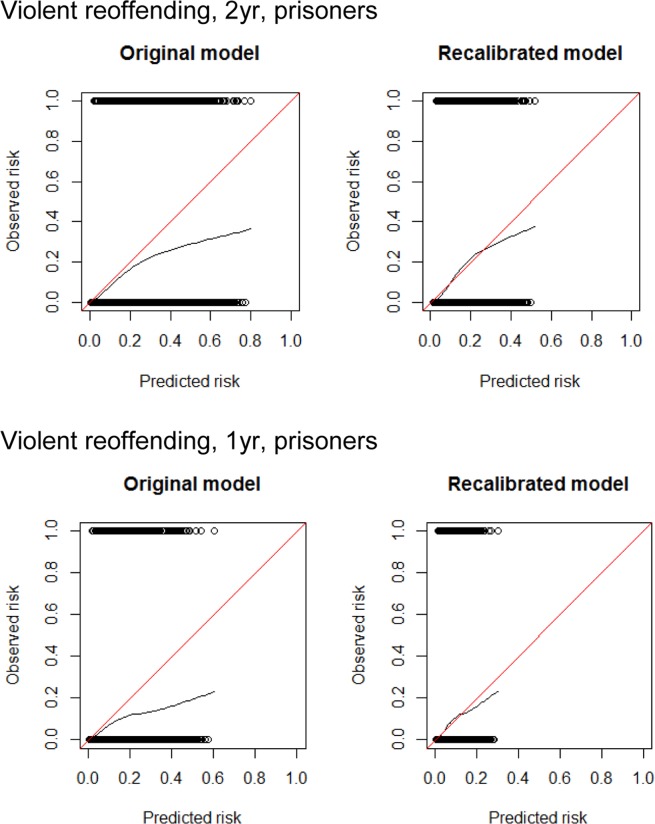
Calibration plots before and after recalibration in prisoners for 1 and 2-year violent reoffending.

Estimates of the baseline risk and multiplicative recalibration shape parameters are shown in Table 3. This procedure improved the calibration of the model substantially, both ‘in the large’ (Supplementary Table 3, final column) and in calibration plots (Fig. 3 and Supplementary Fig. 2), such that that confidence intervals for the ratio of predicted to observed events after recalibration included 1 (Supplementary Table 3) and therefore additional model fitting to obtain new estimates of individual risk factors was not necessary.

**Table 2:** Summary of recalibrated model performance (95% confidence intervals).

	Risk threshold	Prevalence of reoffending	Sensitivity	Specificity	PPV	NPV	c-index (95% CIs)
Violent reoffending, 2 yr, prisoners	10%	16%	91% (89–92)	32% (31–34)	20% (19–22)	95% (94–96)	0.68 (0.66–0.70)
	30%		12% (10–14)	94% (93–95)	27% (23–32)	85% (84–86)	
Any reoffending, 2 yr, prisoners	30%	44%	90% (89–91)	31% (30–33)	51% (50–52)	81% (78–82)	0.69 (0.68–0.70)
	50%		50% (48–52)	74% (73–76)	60% (58–62)	65% (64–67)	
Violent reoffending, 2 yr, non-prisoners	10%	11%	71% (66–74)	59% (58–60)	17% (15–18)	95% (94–95)	0.68 (0.65–0.70)
Any reoffending, 2 yr, non-prisoners	30%	28%	54% (51–56)	69% (68–71)	40% (38–43)	79% (78–81)	0.67 (0.65–0.68)

PPV = Positive predictive value; NPV = Negative predictive value. Note: the 30% (and 50%) threshold was not useful for non-prisoners, as very few had a predicted risk that exceeded this.

**Table 3:** Recalibrated model formulae.

Sweden	Model formula	Baseline risk coefficients	Notes
Violent reoffending	1−S_t_^exp(Σ beta × RF)	S_1_ = 0.7992, S_2_ = 0.6775	
Any reoffending	1−S_t_^exp(Σ beta × RF)	S_1_ = 0.4239, S_2_ = 0.2857	
**The Netherlands**			
Violent reoffending, prisoners	1−S_t_^exp(0.7644 × [−0.0348 × 0.3075 + Σ beta × RF])	S_1_ = 0.8863, S_2_ = 0.7810	*^,†^
Any reoffending, prisoners	1−S_t_^exp(0.8604 × [−0.1275 × 0.3075 + Σ beta × RF])	S_1_ = 0.6447, S_2_ = 0.4450	*
Violent reoffending, non-prisoners	1−S_t_^exp(0.6884 × [−0.0348 × 0.3075 + Σ beta × RF])	S_1_ = 0.9038, S_2_ = 0.8166	*^,†,‡^
Any reoffending, non-prisoners	1−S_t_^exp(0.7741 × [−0.1275 × 0.3075 + Σ beta × RF])	S_1_ = 0.7347, S_2_ = 0.5612	*^,‡^

Notes: ‘beta’ and ‘RF’ refer (respectively) to the model coefficients and risk factors presented in Fazel *et al*.^10^, with certain variables omitted as indicated in the column marked ‘Notes’. The suffix ‘t’ refers to either 1-year risk (t = 1) or 2-year risk (t = 2) in model formulae. The multiples of 0.3075 are adjustments to allow for the immigrant variable being entirely missing in the validation study.

*‘Immigrant’ variable excluded from list of risk factors.

^†^‘Any severe mental disorder’ variable excluded from list of risk factors.

^‡^‘Length of incarceration’ variable excluded from list of risk factor.

Model performance is also expressed in relation to thresholds to define medium and high risk. After recalibration, these were set as 10% and 30% (respectively) for 2-year violent reoffending, and 30% and 50% (respectively) for 2-year any reoffending (Table 2).

## Discussion

This external validation of a scalable violence risk assessment instrument (OxRec) was based on 9072 people in prison and 6329 individuals on probation (‘non-prisoners’). It evaluated violent and any reoffending outcomes at 1 and 2 years according to a prespecified protocol. As it used routinely collected information on predictors and outcomes, this external validation is among the largest in the field of violence risk assessment. Furthermore, it presents findings on a range of performance measures, including discrimination and calibration, rather than selecting those with little practical utility, such as correlation coefficients.

### This study had three main findings

First, we have outlined an approach to recalibrate a prediction model and, in so doing, have demonstrated some optimization of performance. The new model has assumed no difference in the effects of individual predictors compared with the original OxRec model. Second, the recalibrated model may represent a floor of the performance of OxRec in the Netherlands as we relied on proxies for some risk factors. For example, when assessing a history of alcohol use disorder, we relied on a routinely collected item of ‘drinking a lot’, which was present in 21% of prisoners (comparable to 22% who had a history of a diagnosis of alcohol use disorder in the Swedish validation sample) (Table 1). However, for prospective use of OxRec, more precise definitions of predictors should be used, and may lead to different performance. Overall, we report evidence of moderate discrimination for the Dutch version of OxRec, notwithstanding the differences in predictor definitions. This is represented in an overall AUC of 0.68 for 2 year violent reoffending and 0.69 for any reoffending in the prisoner cohort. Other performance measures depend on the risk threshold used (Table 2) with positive predictive values of over 20% and negative predictive values of over 85% for both risk thresholds of 10% and 30% violent reoffending risk, and positive predictive values of over 50% for any reoffending risk. A final finding was the performance of the model was not materially inferior when we tested it in non-prisoners – with AUC values of 0.68 and 0.67 for 2-year violent and any reoffending, respectively – although risk estimates needed to be adjusted downwards in recalibration to reflect the lower base rate of reoffending in this population.

Compared to the most common violence risk assessment tools, the reduction in discrimination was less in this external validation of OxRec than in validations of other instruments. Recent field studies of the HCR-20 in Belgium have reported an AUC of 0.60 in forensic psychiatry^11^, and for the PCL-R, it was 0.55 in released prisoners^12^. In Scotland, a validation study of the HCR-20 reported an AUC of 0.60 in released forensic psychiatric patients^17^, and 0.62 for the short version of the PCL in the same population^18^. These represent larger reductions in discrimination from around 0.70 in predominately small research studies^16^. In selected populations of high risk prisoners, the HCR-20 has performed better^19^, which reflects higher base rates of reoffending, although the performance of the PCL-R was worse^20^. A recent review of US studies looking at recidivism from correctional settings, and therefore similar to this study, found that in the 4 field studies of the LSI-R, the AUC was 0.63 (IQR 0.60–0.66)^21^. Other tools used in similar samples were the RMS (Risk Management System) and the WRN (Wisconsin Risk and Needs), with AUCs of 0.66 and 0.67, respectively that included the calibration samples, with poorer performance in probation samples for the WRN^22^. In the current study, the false positive rate was high when the lower threshold of 10% was used (e.g. 68% for violent reoffending in prisoners at 2 years), although this is not different from other violence risk assessment tools (where the pooled rate was 64%^16^). However, at the 30% threshold for 2 year violent reoffending in prisoners, the false positive ratio was 6%.

### There are three main implications to this study

First, the recalibrated version of OxRec can be used for individuals on release from prison in the Netherlands, and also appears to have adequate measures of discrimination for people on probation. Second, we have demonstrated that it is possible to recalibrate prediction models and retain adequate performance. Our methods provide an approach to do so by using an incremental strategy of adjusting the baseline risk, and then recalibrating the linear predictor component of the prediction tool. To our knowledge, risk assessment tools are currently being used in new samples without any recalibration, which will likely detract from performance. However, if it is found that the effects of individual predictors in the new sample is very different from the original model, then recalibration alone is unlikely to be sufficient and is therefore not recommended, but rather a new derivation study may be required. Third is the importance of prospective validation, in which individual predictors can be aligned more closely with those in the risk assessment tool being tested. This is currently ongoing in the Netherlands and provide triangulation of the replication. Comparing its performance against other measures, such as the RISc that has been used by Dutch Probation Services, could also be considered.

Strengths of this study include the large sample size of around 15,000 individuals, the representativeness of the sample due to the use of total population dataset of all individuals who were convicted of crimes, the prespecification of a protocol, and the presentation of a range of performance metrics.

However, there are some weaknesses, partly due to the use of routinely collected data that did not align with the exact definitions of the predictors in the original OxRec study. Variations in the characteristics of the samples (Table 1) and these definitions may have contributed to the different performance of the Dutch validation compared to the Swedish one. Other limitations include that OxRec contains up to four modifiable factors, which means that treatment matching is mostly focused on substance use and other psychiatric disorders. Further, some items are not easily generalizable to other countries, and will require modification, such as educational level and neighbourhood deprivation score. Another limitation is that the tool provides a cross-sectional assessment at one time point (on release from prison), and therefore cannot be used to monitor risk in the community. Tools with more dynamic factors, where changes in risk scores can improve prediction, should be considered for risk monitoring^23^. The risk categories used in this study may not be suitable in other countries or criminal justice populations, and each new validation should consider using categories aligned to expected reoffending rates. The probability scores in OxRec avoids this potential limitation. Finally, some potentially important predictors were not tested as they are not captured in routinely collected datasets, but may add incremental validity and allow for improved treatment matching.

In summary, we have presented the external validation of a scalable risk assessment tool for individuals convicted of crimes, which supports its translation into routine practice. Other jurisdictions implementing risk assessment instruments should review their performance, and consider optimising calibration using the approach we have outlined.

## Methods

### Data sources

Data were obtained from the Research and Documentation Centre (WODC) of the Ministry of Justice in the Netherlands^24^, Statistics Netherlands (CBS)^25^ and the RISc Database of the Dutch Probation Services (3RO)^26^. The RISc (Recidivism Assessment Scales) is the tool used by Dutch Probation for screening offenders.

The retrospective study cohort was separated into two groups that were evaluated independently. These consisted of those individuals released from prison (‘prisoner cohort’) and non-prisoners who were undergoing a probationary risk assessment following one or more previous convictions (‘non-prisoner cohort’), respectively. Both groups used risk assessments taking place in the Netherlands in 2011 or 2012 so that complete two-year follow-up data on all participants could be obtained. The prisoner cohort was as close as possible to the original Swedish cohort, and the non-prisoner cohort allowed us to additionally check whether OxRec’s performance was equally good in a lower-risk group.

### Prediction model

The objective was to perform an external evaluation (and, if necessary, updating) of OxRec. This original model was developed and externally validated in a cohort of 47,326 individuals released from prison in Sweden between 2001 and 2009, is available as a free web-based calculator (https://oxrisk.com/oxrec), and its protocol published (https://ars.els-cdn.com/content/image/1-s2.0-S2215036616001036-mmc1.pdf).

### Definition of outcomes

Outcome and predictor variables were defined to match the definitions used in the original OxRec study as closely as possible. The primary outcome was violent crime conviction within 12 months and 24 months in the prisoner cohort. Violent crime was defined as any conviction for any violent offence, including sexual offences and robbery. Reoffending for any crime (violent or non-violent) was a secondary outcome. Evaluation of both outcomes in the non-prisoner cohort, to test the performance of the OxRec in a lower-risk group, was an additional secondary objective. Time until offence was measured from an index date of either the release date (in the prisoner cohort) or the time of the risk assessment (in the non-prisoner cohort).

### Definition of predictors

Differences in recording practices between Sweden and the Netherlands required clarification in the definitions of some predictor variables compared to the definitions in the original study^10^, as described in Supplementary Table 1. No new predictors were used. For one predictor (neighbourhood deprivation), a principal component analysis based on 5 postcode-level variables was used to develop a score based on deciles (Supplementary Table 1). The original OxRec used a similar analysis based on 8 postcode-level variables^27^.

### Statistical methods

A full statistical analysis plan is available as Supplementary Material. We evaluated the performance of the OxRec model in predicting 1-year and 2-year risk separately in the prisoner cohort and the non-prisoner cohort. Prediction performance is presented in terms of measures of discrimination (c-index, or area under the receiver operating characteristic (ROC) curve) and calibration, both ‘in the large’, i.e. in terms of the total number of offences predicted, and via calibration plots^28^.

We initially evaluated model performance without modifying OxRec for the new population. In the case of inadequate calibration, our approach was to follow a conservative incremental strategy that has been suggested previously^29,30^. This involved (i) performing simple validation of the existing OxRec model applied to the Dutch dataset and then, if calibration was inadequate, subsequently (ii) updating the baseline risk, without changing the coefficients of predictor variables, and if necessary (iii) additionally re-calibrating the coefficients of the predictors via a single multiplicative recalibration parameter^31^. Although the analysis plan also allowed for a further step, (iv) re-estimation of the coefficients of individual predictors, we preferred to avoid this as the objective was primarily validation rather than to create a new prediction tool, and it transpired that steps (i)-(iii) were sufficient to achieve adequate calibration. Re-estimation was therefore not necessary.

Entirely missing predictors were either reset to zero for all participants (‘any mental health disorder’), on the basis that it was rare, or to the prevalence level in the Swedish cohort (‘immigrant status’ as this predictor was not extracted). As this decision has the effect of adding a constant to the linear predictor for all participants, it becomes unimportant in models for which the baseline risk needs to be updated. For predictors that were missing in only a subset of individuals, we used multiple imputation with chained equations and estimated model performance across 20 imputations^32^. We used similar multiple imputation methods and models to estimate the recalibration parameter.

We used R version 3.2.4 (R Core Team, 2016) for all analyses^33^ and followed published guidance on reporting validation study results^34–36^.

### Deviations from protocol

We indicated the outcomes, predictors, risk categories and analytic plan in a protocol before data analysis (‘prespecified protocol’). We initially evaluated the performance of OxRec in relation to the probability thresholds to define low or high risk that were specified in the original paper. After recalibration, it became apparent that using the same set of thresholds in the Dutch cohort would not be useful, because of systematic differences in levels of risk and the distribution of the risk factors in this population. In these cases, we therefore present a revised set of probability thresholds in the sample to aid interpretation. These new thresholds had a similar prevalence in each threshold than the original OxRec model (being rounded up or down to the nearest 10% to simplify use). Sensitivity, specificity, positive and negative values associated with these thresholds were calculated, with 95% confidence intervals. The actual sample size of this validation study was more than 10-fold larger than the original estimates reported in the protocol (where it was stated as 792 prisoners and 798 probationers) due to national data becoming available.

Statistics Netherlands (CBS), Research and Documentation Centre (WODC) of the Ministry of Justice in the Netherlands and the Probation Services (3RO) granted approval to use the data for this study, which was anonymized.

## Supplementary information

Supplementary information
