# Supplementary material for: Prediction of violent reoffending in prisoners and individuals on probation: a Dutch validation study (OxRec)
Source: Sci Rep. 2019 Jan 29;9:841. doi: 10.1038/s41598-018-37539-x (PMC6351626; doi:10.1038/s41598-018-37539-x)
Supplement: Supplementary file 1 — Supplementary information [file 41598_2018_37539_MOESM1_ESM.pdf]

Prediction of violent reoffending in prisoners and individuals on probation: a Dutch validation study (OxRec)

Seena Fazel, Achim Wolf, Maria Vazquez Montes, Thomas R Fanshawe

Supplementary Table 1. Variable definitions

Variable	Sweden	Netherlands
Sex	Assigned at birth	Same definition
Age	Age at release from prison	Same definition
Immigrant	First or second generation immigrants (self or either parent born outside of Sweden)	Not included
Length of incarceration	Duration of incarceration for most recent offence	Same definition. Non-prisoners were assigned to the lowest category (<6 months)
Violent index offence	Most recent offence was homicide, assault, robbery, arson, any sexual offence (rape, sexual coercion, child molestation, indecent exposure, or sexual harassment), illegal threats, or intimidation.	Most recent offence was a violent offence, including sexual offences and robbery
Previous violent crime	Any conviction for a violent offence previous to most recent offence (i.e. Before index offence).	Any conviction for a violent offence as defined above, previous to most recent offence.
Civil status	Unmarried vs other (At imprisonment. Other includes married, cohabiting, divorced, and widowed)	Single (including single parent and living unmarried with parents or others) vs. Other (living together, married)
Education	Lower secondary, upper secondary, post-secondary	'Primary or special education', 'No secondary diploma' and 'Secondary diploma'.
Employment	Employed at incarceration. (Worked for at least 4 hours [based on their income information] during November before incarceration)	"Employed or useful daily occupation"
Income	Negative/Zero/Low/Medium/High (Low: <20thpercentile, Medium: 20-80thpercentile, High: >80thpercentile). 'Low' and 'Medium' disposable income had accounted for 93%.	'Medium' = "stable, healthy financial situation" 'Low' = otherwise. Other (minority) income categories could not be classified and so were not used.
Deprivation	Principal components analysis of: mean disposable income, % welfare recipients, % unemployed, % divorced individuals, % with only primary school qualifications, % of immigrants (defined as individuals who were not born in Sweden), residential mobility rate, and crime rate.	Principal components analysis of five postcode-level variables: % welfare recipiency, % unemployed, % low education, crime rate, median income
Alcohol use	Diagnosis of alcohol use disorder (lifetime: before or during incarceration – ICD-8: 291, 303; ICD-9: 291, 303, 305A; ICD-10: F10).	Individuals recorded as "drinking a lot", either 'currently' or 'in the past'

Drug use	Diagnosis of drug use disorder (lifetime: before or during incarceration – ICD-8: 304; ICD-9: 292, 304, 305 excl. 305A; ICD-10: F11-F19).	Using any of "heroin, cocaine, opiates or speed", "at least three times per week"
Any mental disorder	Diagnosis of any mental disorder excluding substance use disorders (lifetime: before or during incarceration).	Those classified as having "psychiatric problems over a long period".
Any severe mental disorder	ICD diagnosis of schizophrenia-spectrum or bipolar disorder (lifetime: before or during incarceration).	Not included

Note: Items in the Netherlands were extracted from the RISC database

Outcomes	Sample of prisoners (n=9072)	Sample of non-prisoners (n=6329)	Comparison with Fazel et al. (2016)
2yr violent reoffending	1445 (16%)	670 (11%)	21%
1yr violent reoffending	747 (8%)	346 (5%)	12%
2yr any reoffending	3967 (44%)	1795 (28%)	59%
1yr any reoffending	2504 (28%)	1051 (17%)	44%

Supplementary Table 2. Summary of outcomes in prisoners and non-prisoners for the Dutch and original OxRec samples.

	Observed number of events	Predicted number of events (uncalibrated)	Ratio, Predicted: Observed (uncalibrated) (95% CI)	Ratio of crude event rates, Sweden: Netherlands	Predicted number of events (after recalibration)	Ratio, Predicted: Observed (after recalibration) (95% CI)	Brier score
Violent reoffending, 2yr, prisoners	1098	1524	1.39 (1.31, 1.47)	1.29	1087	0.99 (0.93, 1.05)	0.129
Violent reoffending, 1yr, prisoners	561	952	1.70 (1.56, 1.84)	1.43	563	1.00 (0.92, 1.09)	0.074
Any reoffending, 2yr, prisoners	3130	3981	1.27 (1.23, 1.32)	1.33	3131	1.00 (0.97, 1.04)	0.221
Any reoffending, 1yr, prisoners	1971	3160	1.60 (1.53, 1.68)	1.61	1978	1.00 (0.96, 1.05)	0.185
Violent reoffending, 2yr, non-prisoners	537	809	1.51 (1.38, 1.64)	1.95	536	1.00 (0.92, 1.09)	0.090
Violent reoffending, 1yr, non-prisoners	277	495	1.79 (1.58, 2.01)	2.16	278	1.00 (0.89, 1.13)	0.049
Any reoffending, 2yr, non-prisoners	1488	2348	1.58 (1.50, 1.66)	1.56	1483	1.00 (0.95, 1.05)	0.188
Any reoffending, 1yr, non-prisoners	865	1803	2.08 (1.95, 2.23)	2.67	867	1.00 (0.94, 1.07)	0.131

Supplementary Table 3. Calibration ‘in the large’: Observed and predicted number of events using the original OxRec tool in the Dutch sample, event rate comparison with the Swedish cohort, and predicted number of events after recalibration in the Dutch sample.

Note: Results here are presented for individuals with complete risk factor data. No data were available for Swedish non-prisoners, so the ratios of crude event rates are in all cases calculated relative to the rates in Swedish prisoners.

(References to calculate the CIs: Hanson, R. K. (2017). Assessing the calibration of actuarial risk scales: A primer on the E/O index. Criminal Justice and Behavior, 44(1), 26-39; Rockhill B, Byrne C, Rosner B, Louie MM, Colditz G (2003). Breast cancer risk prediction with a log-incidence model: Evaluation of accuracy. J Clin Epid 56: 856-861.)

Supplementary Figure 1

Receiver operating characteristic curve for 1-year violent reoffending in Dutch prisoners and non-prisoners.

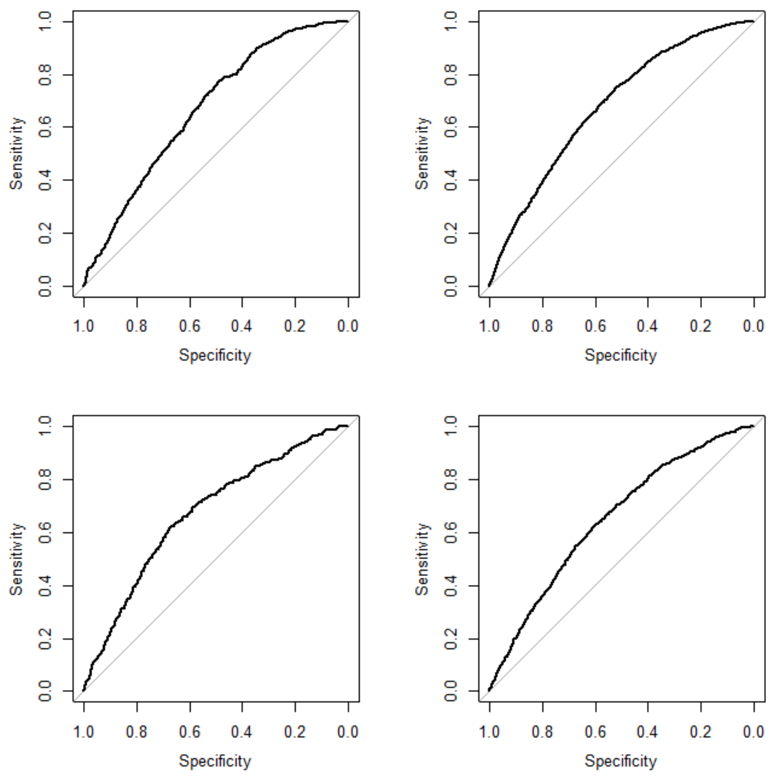

Note: Upper left: 1-year violent reoffending, prisoners. Upper right: 1-year any reoffending, prisoners. Lower left: 1-year violent reoffending, non-prisoners. Lower right: 1-year any reoffending, non-prisoners.

Violent reoffending, 2yr, prisoners

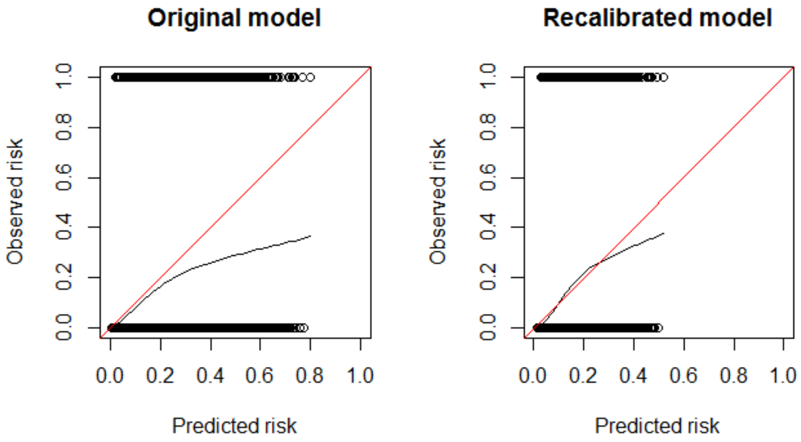

Violent reoffending, 1yr, prisoners

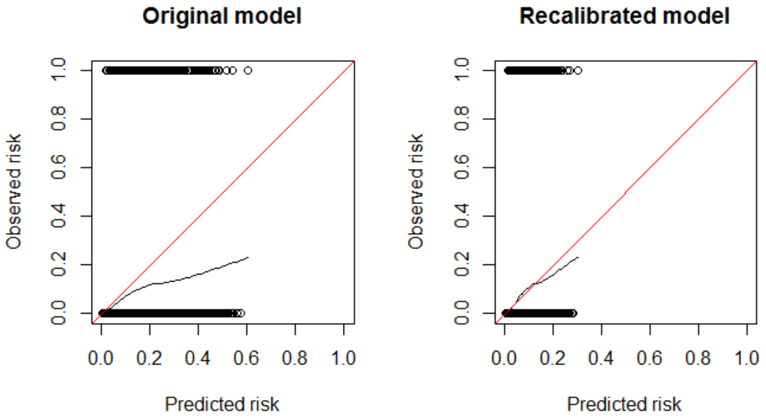

Any reoffending, 2yr, prisoners

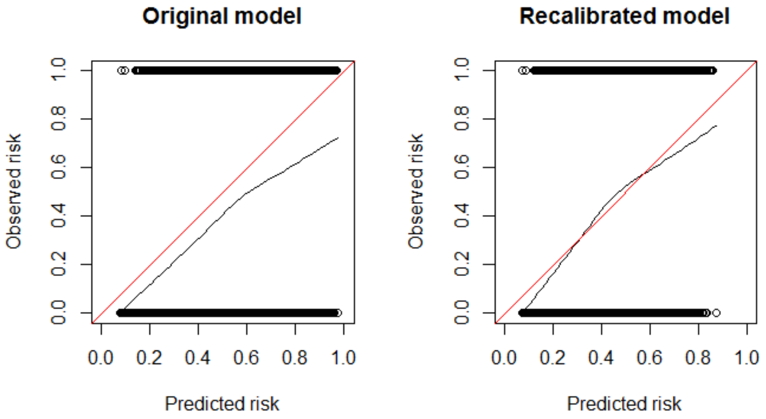

Supplementary Figure 2a: Calibration plots before and after recalibration for prisoners

Any reoffending, 1yr, prisoners

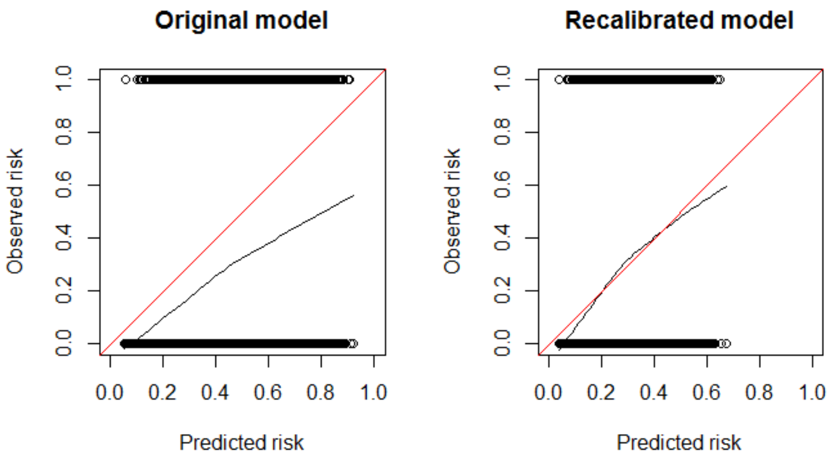

Violent reoffending, 2yr, non-prisoners

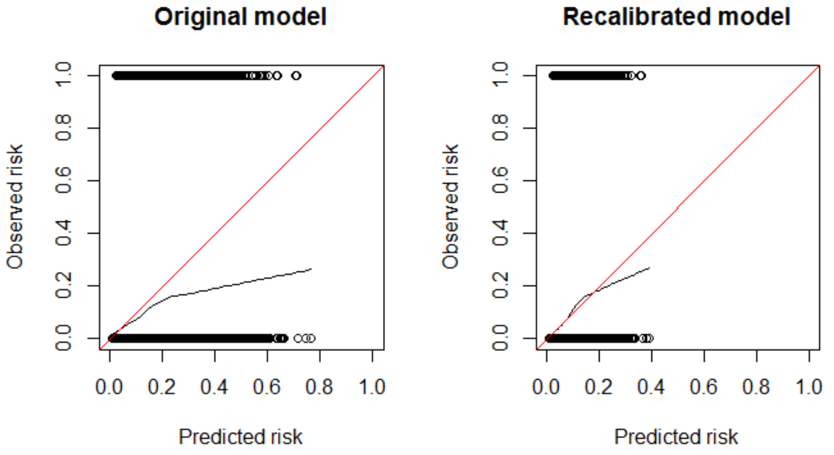

Violent reoffending, 1yr, non-prisoners

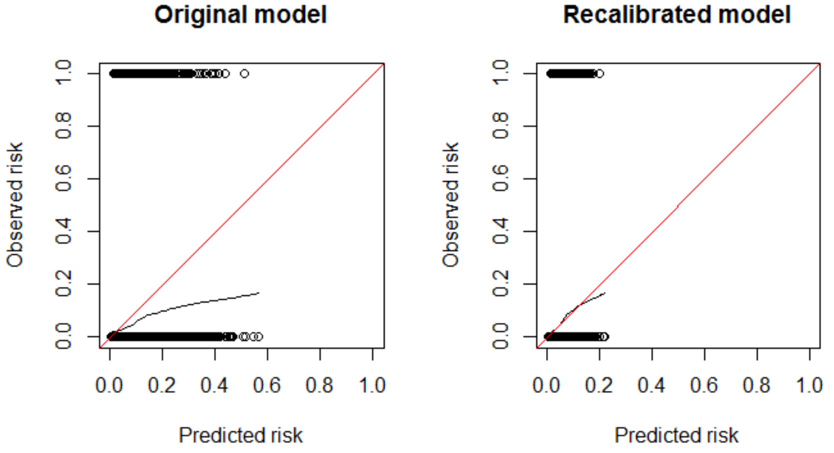

Any reoffending, 2yr, non-prisoners

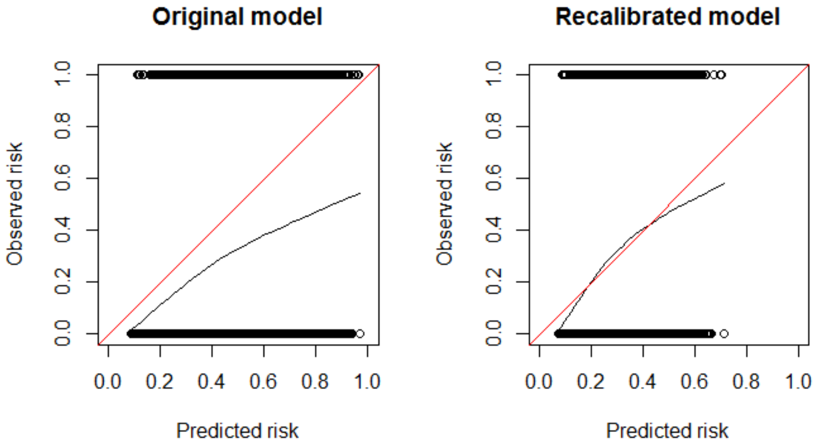

Any reoffending, 1yr, non-prisoners

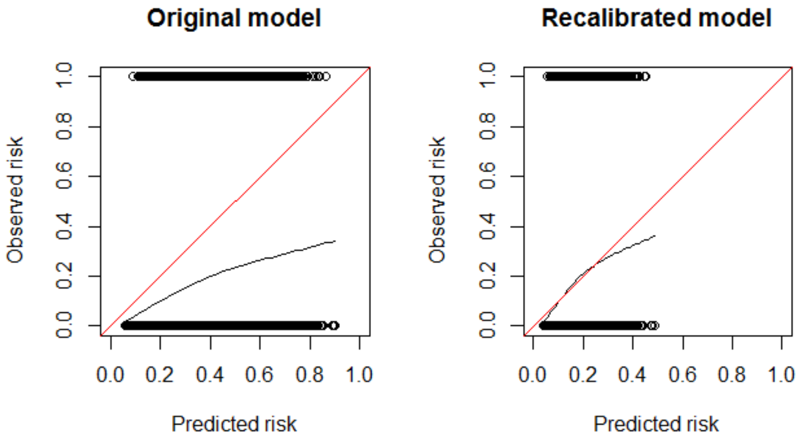

Supplementary Figure 2b: Calibration plots before and after recalibration for non-prisoners

Validation of OxRec (violent reoffending on release from prison) – statistical analysis plan

Background and Study Summary

The OxRec (Oxford Risk of Recidivism) tool for predicting violent reoffending on release from prison was published in 2016, based on data from a Swedish cohort (Fazel et al., 2016). It is a Cox proportional hazard model based on a 14-item panel of risk factors, encompassing socio-demographic, criminal history and clinical variables.

A web calculator has also been published (https://oxrisk.com/oxrec). Upon entry of the 14 risk factors, this calculator estimates the probability of re-offending, expressed as a percentage, within 12 months and within 24 months. A categorical classification (risk level) of 'low' (probability < 10%), 'medium' (10-50%) or high (> 50%) based on the 24-month predictive probability is also assigned. Certain risk factors are permitted to be missing ('unknown'). In this case, the calculator returns a range of predictive probabilities corresponding to the maximum and minimum that would be possible to obtain had the risk factor been available. If this range spans more than one risk level, all indicated risk levels are displayed.

The objective of the current study is to assess the predictive performance of OxRec using data from an external cohort.

Data are available from prison and probation reports from the Netherlands, from 2012, with two-year follow-up. Participants are individuals undergoing either (i) prison or (ii) probationary (non-prison) assessment at time of release from prison.

Outcomes are

(i) Violent reoffending within 12 months of release;

(ii) Violent reoffending within 24 months of release.

Data

For the validation study, data are available from approximately equal numbers of prisoners (792) and (probationary) non-prisoners (798) (cf. a cohort of 47,326 prisoners in the Swedish development dataset).

Sample size

Preliminary data suggest a total sample size of 792 prisoners, with 88 (11%) violent crime events within 12 months and 154 (19%) violent crime events within 24 months. In addition, data are available from 798 non-prisoners (approximately 133 events) (see section 'Cohort definition' below). These figures appear comparable to estimated probabilities of reoffending of 11% and 18% within 1 year and 2 years respectively in the Swedish development dataset.

A simulation study from 2005 recommended ballpark minimum figures of 100 events for validating a clinical prediction rule derived using a logistic regression model (Vergouwe et al., 2005). These conclusions were broadly supported by another, more recent, simulation study (Collins et al., 2016b). In practice, the sample size of the current study is limited by data availability, and in particular by the number of recurrent violent crime events within the maximum two-year follow-up period. As the objectives of the study include both validation and possible updating of the tool for the Dutch cohort if validation is found to be poor, no formal sample size calculation is performed.

Statistical Analysis

Diagnostic performance measures

The analysis will evaluate the calibration and discrimination of the model (Collins et al., 2016a). Calibration is a measure of how close the predicted outcomes are to the observed outcomes. This measure is usually evaluated graphically through calibration plots which compare the relationship observer-predicted outcomes to a diagonal line. Discrimination is a measure of how well the model distinguishes between individuals that committed a criminal re-offense and those who did not. The most common measure of discrimination is the c-index which can be adapted to survival models (Pencina et al., 2012). A list of the specific measures employed in this analysis is presented below (see section 'Presentation of results').

Strategy for analysis

Our approach to assessing the diagnostic performance of OxRec in the Dutch cohort will follow an incremental strategy that has been suggested previously (Steyerberg, 2009; Su et al., 2016). Details relating to the validation of survival models are also available (van Houwelingen, 2000). We will begin by assessing individual-level diagnostic performance with simple validation of the existing model in mind, without any adjustment of the model coefficients for the new population (Royston and Altman, 2013). If diagnostic performance is poor, we will consider a

series of conservative updating steps to investigate the feasibility of adapting the prediction model to the Dutch population.

Specifically, we will carry out the following steps, proceeding in each case to the subsequent stage only if diagnostic performance is insufficient. For example, if the first (simple validation) stage indicates adequate performance, the study will be treated solely as a validation study and no additional updating will be performed. We anticipate making the assessment of diagnostic performance between steps (i) to (iv) below based on statistical significance (retaining the simplest version model for which the subsequent step provides no statistically significant improvement) and the final assessment of diagnostic performance based on clinical utility – in particular in relation to the equivalent measures reported in the model development paper (Fazel et al., 2016).

(i) Simple validation: assess the diagnostic performance of the existing OxRec model directly using the Dutch cohort data. This means applying the existing model directly, without adjusting model coefficients and without adjusting the estimate of baseline risk. Calibration will be assessed both 'in-the-large' (i.e. to check whether the model adequately estimates the prevalence of events across the whole cohort) and at individual level using calibration plots and the estimated calibration slope (Steyerberg, 2009). Discrimination will be assessed using summary statistics similar to those reported in the original study, including the c-index.

(ii) Updating the baseline risk (analogous to the intercept), holding coefficients of predictor variables at their values from the current model. This may also be seen as adjusting for any systematic risk difference that may be attributable to missing covariates (see subsection below).

(iii) Updating the baseline risk and performing a simple re-calibration of the coefficients of the predictors. This allows for the inclusion of a single re-calibration parameter, i.e. the effect of predictors on the outcome would be assumed to be the same as in the original study, apart from rescaling.

(iv) Additionally re-estimating a subset of the model coefficients. This will be performed only as a last resort as it is likely to lead to a substantively different prediction tool and would allow the effect of risk factors on the outcome to differ from those found in the original study. If possible, re-estimation will be restricted to a subset of parameters whose coefficients appear to be poorly calibrated, as the current study is unlikely to have a high enough event rate to re-estimate all parameters and is not intended to be a model development study.

Cohort definition

The Dutch cohort contains approximately equal numbers of prisoners and probationer non-prisoners. The original study was intended to be used upon release from prison; therefore primary validation will be performed and reported based on the Dutch prisoner cohort. Brief assessment will also be made in the non-prisoner cohort to test transportability into a group with qualitatively different characteristics.

Missing predictors and missing data

We anticipate that most predictor variables, and in particular the stronger predictors of reoffending, will be complete or substantially complete in the Dutch cohort. The variable 'severe mental illness' is likely to be either entirely missing or to be based on a significantly different definition from the one used in the original study. We will investigate the impact of imputing this low-prevalence variable in two ways: firstly, by setting it to zero in all participants (a form of mean imputation), and secondly, if required, using multiple imputation assuming the association with other variables is similar to that observed in the Swedish cohort.

The 'immigrant' variable is required to be omitted from the tool for use in the Dutch cohort. This variable will therefore be treated as if missing for the purpose of validation. We plan to assign an average value to this variable for all participants, equivalent to its effect being incorporated into the model intercept.

Multiple imputation within the Dutch cohort alone will also be considered if there are other variables with non-negligible quantities of missing values (more than 20%). It is not possible to use multiple imputation, based on the Dutch data by itself, for variables that are entirely missing.

It is acknowledged that using multiple imputation is unlikely to reflect the use of the OxRec tool in practice, as the web calculator requires missing variables to be either arbitrarily set at a particular assumed value or (in some cases) to be retained as missing, in which case a range of prediction is presented. For this reason we will additionally consider a worst-case/best-case scenario analysis using the upper and lower limit of the probability estimate generated by the risk calculator. Further information on dealing with missing predictors in validation studies has been previously published (Held et al., 2016)

New predictors

The study will use the 14 predictor variables that make up the OxRec risk tool. No new predictors are available. It is acknowledged that some variable definitions may vary slightly

between settings. In this case the variables have been chosen that are thought to match most closely with those used in the OxRec tool.

Censoring

In the original study, all individuals in the development cohort were used for model fitting, excluded individuals who were censored before the end-point without having had the outcome of interest were excluded for the purpose of summarising model performance. If the censoring in the validation sample is substantial, we will compare this approach to the Chambless/Diao estimator described in a recent methodological review on this subject (Kamarudin et al., 2017).

Predicted probabilities and risk categories

The current version of the web calculator presents both probabilistic results and two-year risk expressed as a risk category (‘low’, ‘medium’ or ‘high’). In the validation study results will be primarily presented based on probabilities, as this is likely to be the more sensitive measure for detecting patterns of miscalibration. However, predictive performance of the two-year risk category will also be assessed using contingency tables and summary statistics.

Presentation of results

Assessment will be presented using both calibration and discrimination, graphically and using tables and summary statistics as appropriate. These will include calibration plots together with estimated calibration intercept and slope; sensitivity and specificity; Brier score; c-index.

The report will include descriptive information about the cohort and how it was defined, including any differences from the original cohort in definitions of variables. As far as possible we will follow published guidance on reporting validation study results (Bouwmeester et al., 2012; Collins et al., 2014; TRIPOD Group).

Date: 2nd August 2017

References

Bouwmeester, W., Zuithoff, N. P. A., Mallett, S., Geerlings, M. I., Vergouwe, Y., Steyerberg, E. W., Altman, D. G. & Moons, K. G. M. (2012). Reporting and Methods in Clinical Prediction Research: A Systematic Review. *PLOS Medicine,* **9**, e1001221.

Collins, G., Ma, J., Gerry, S., Ohuma, E., Odondi, L. O., Trivella, M., Beyer, J. & Vazquez-Montes, M. (2016a). Risk prediction models in perioperative medicine: Methodological considerations. *Curr Anesthesiol Rep*.

Collins, G. S., de Groot, J. A., Dutton, S., Omar, O., Shanyinde, M., Tajar, A., Voysey, M., Wharton, R., Yu, L.-M., Moons, K. G. & Altman, D. G. (2014). External validation of multivariable prediction models: a systematic review of methodological conduct and reporting. *BMC Medical Research Methodology,* **14**, 40.

Collins, G. S., Ogundimu, E. O. & Altman, D. G. (2016b). Sample size considerations for the external validation of a multivariable prognostic model: a resampling study. *Statistics in Medicine,* **35**, 214-226.

Fazel, S., Chang, Z., Fanshawe, T., Långström, N., Lichtenstein, P., Larsson, H. & Mallett, S. (2016). Prediction of violent reoffending on release from prison: derivation and external validation of a scalable tool. *The Lancet Psychiatry,* **3**, 535-543.

Held, U., Kessels, A., Garcia Aymerich, J., Basagaña, X., ter Riet, G., Moons, K. G. M. & Puhan, M. A. (2016). Methods for Handling Missing Variables in Risk Prediction Models. *American Journal of Epidemiology,* **184**, 545-551.

Kamarudin, A. N., Cox, T. & Kolamunnage-Dona, R. (2017). Time-dependent ROC curve analysis in medical research: current methods and applications. *BMC Medical Research Methodology,* **17**, 53.

Pencina, M., D’Agostino, R. & Song, L. (2012). Quantifying discrimination of Framingham risk functions with different survival C statistics. *Stat Med. ,* **31**, 1543–53.

Royston, P. & Altman, D. G. (2013). External validation of a Cox prognostic model: principles and methods. *BMC Medical Research Methodology,* **13**, 33.

Steyerberg, E. (2009). *Clinical Prediction Models.* Springer.

Su, T.-L., Jaki, T., Hickey, G. L., Buchan, I. & Sperrin, M. (2016). A review of statistical updating methods for clinical prediction models. *Statistical Methods in Medical Research*.

TRIPOD Group. *TRIPOD checklist*. URL: http://www.tripod-statement.org/TRIPOD/TRIPOD-Checklists.

van Houwelingen, H. C. (2000). Validation, calibration, revision and combination of prognostic survival models. *Statistics in Medicine,* **19**, 3401-3415.

Vergouwe, Y., Steyerberg, E. W., Eijkemans, M. J. C. & Habbema, J. D. F. (2005). Substantial effective sample sizes were required for external validation studies of predictive logistic regression models. *Journal of Clinical Epidemiology,* **58**, 475-483.

Appendix: definition of variables

The table refers to the variables as defined in the Swedish cohort.

Variable	Type	Notes
Sex	Binary	Male / female
Age*	Continuous	Age at release (allowed range: 16-75)
Immigrant	Binary	First / second generation immigrant (born outside Sweden)
Length of incarceration	Categorical	For most recent offence<br>< 6 months<br>6-12 months<br>12-24 months<br>≥ 24 months
Violent index offence	Binary	Most recent offence was homicide, assault, robbery, arson, any sexual offense (rape, sexual coercion, child molestation, indecent exposure, or sexual harassment), illegal threats, or intimidation
Previous violent crime (before index offence)	Binary	Previous to most recent offence
Civil status*	Binary	At time of imprisonment<br>Unmarried<br>Other (including married, cohabiting, divorced, and widowed)
Highest education*	Categorical	Up to age 16<br>Age 16-18<br>Beyond age 18
Employment	Binary	At time of incarceration
Disposable income*	Categorical	Negative<br>Zero<br>Low (< 20th percentile)<br>Medium (20th-80th percentile)<br>High (≥ 80th percentile)
Neighbourhood deprivation*	Categorical	Deciles (1 – lowest deprivation to 10 – highest deprivation)
Alcohol abuse	Binary	Diagnosis of alcohol use disorder before or during incarceration
Drug abuse	Binary	Diagnosis of drug use disorder before or during incarceration

Any mental disorder	Binary	Diagnosis of any psychiatric disorder (except alcohol or drug use disorder) before or during incarceration
Any severe mental disorder	Binary	Diagnosis of schizophrenia-spectrum or bipolar disorder before or during incarceration

* indicates that variable may be entered as a missing value
